# The roles of dispersal limitation and pre-adaptation in shaping *Paraburkholderia* endosymbiont frequencies in social amoeba communities

**DOI:** 10.1128/aem.01615-25

**Published:** 2025-11-07

**Authors:** James G. DuBose, Terry Uhm, Jordan Bowen, Patricia Fiedorek, Mackenzie Hoogshagen, Tamara S. Haselkorn, Susanne DiSalvo

**Affiliations:** 1 Department of Biology, University of Central Arkansas 2673, Conway, Arkansas, USA; 2 Department of Biology, Emory University 1371, Atlanta, Georgia, USA; 3 Department of Biological Sciences, Southern Illinois University Edwardsville 33140, Edwardsville, Illinois, USA; Georgia Institute of Technology, Atlanta, Georgia, USA

**Keywords:** dispersal, endosymbiosis, *Paraburkholderia*, dictyostelid, social amoeba

## Abstract

Endosymbiotic interactions have long played fundamental roles in shaping the evolution and diversification of eukaryotes. However, we still have a limited understanding of how ecological processes govern the distribution of endosymbionts that are still segregating in host populations. To contribute to this understanding, here, we use the interactions between *Paraburkholderia* endosymbionts and their dictyostelid social amoeba hosts as a model system to investigate the role of dispersal, a fundamental ecological process, in shaping the distribution and evolution of endosymbiotic interactions. We first found that patterns of endosymbiont diversification were highly biogeographic, suggesting a significant degree of dispersal limitation. We then experimentally mediated the dispersal of several endosymbiont species into environments with multiple host species and found that each symbiont was able to sustain a high prevalence in each host population. The benefit/detriment of these mediated interactions did not change with increasing phylogenetic distance from what is suspected to be the focal amoeba host species in nature. Taken together, our findings suggest *Paraburkholderia* endosymbionts are generally pre-adapted to occupy a variety of dictyostelid host environments, and their distribution among host populations is subject to a high degree of dispersal limitation. Overall, our findings have significant implications for our understanding of how ecological processes facilitate and limit the evolution of endosymbiotic interactions.

**IMPORTANCE:**

Endosymbiotic interactions are ubiquitous in complex eukaryotes, as organelles such as mitochondria and chloroplasts represent the remnants of what were once free-living prokaryotes. However, how ecological processes facilitate the transition from free-living to host-associated is less understood. Selection is the most commonly invoked process to explain this transition: symbionts that are better at infecting hosts and potentially confer some benefit rise in frequency because they are selected for (and otherwise selected against). However, this only describes one fundamental process that can shape the ecology of symbiotic interactions. Here, we present evidence that the importance of dispersal (and its limitations) likely exceeds that of selection in shaping the distribution and frequency of *Paraburkholderia* endosymbionts in their dictyostelid social amoeba host communities. These findings highlight the need to consider regional ecological processes that operate at a scale beyond the individual when studying ecology and evolution of endosymbiotic interactions.

## INTRODUCTION

It is difficult to understand the evolution of complex eukaryotes without considering the endosymbiotic interactions they have formed and maintained throughout natural history. The most notable examples of this point are mitochondria and chloroplasts, which have fundamentally shaped the evolution of eukaryotes (1). More generally, other endosymbioses have independently arisen that have significantly shaped the evolution and diversification of many lineages (2–5). Notable progress has been made in understanding the molecular and genetic bases underlying a symbiont’s evolutionary transition to an endosymbiotic lifestyle, as well as the macroevolutionary significance to their host’s lineage (6). However, how ecological processes govern the distribution of endosymbionts that are still segregating in host populations has received relatively less empirical attention.

Ecological theory describes the process that shapes the distributions and frequencies of organisms as analogous to those that shape the distributions and frequencies of alleles: selection, drift, dispersal (gene flow), and diversification (mutation) (7). From both an ecological and evolutionary perspective, the primary process ascribed to explain the distribution of symbionts among host taxa is selection: selective pressures exerted by or on hosts facilitate or eliminate symbiont residence, which increases host fitness (8). However, ecological theory recognizes the interplay between different processes and aims to understand the relative importance of each processs in shaping the distributions of organisms. Only recently has investigation into the distribution of host-symbiont interactions begun to shift from a selection-centric framework to one that focuses on integrating more general ecological processes, such as ecological drift and dispersal (9).

**Fig 1:**
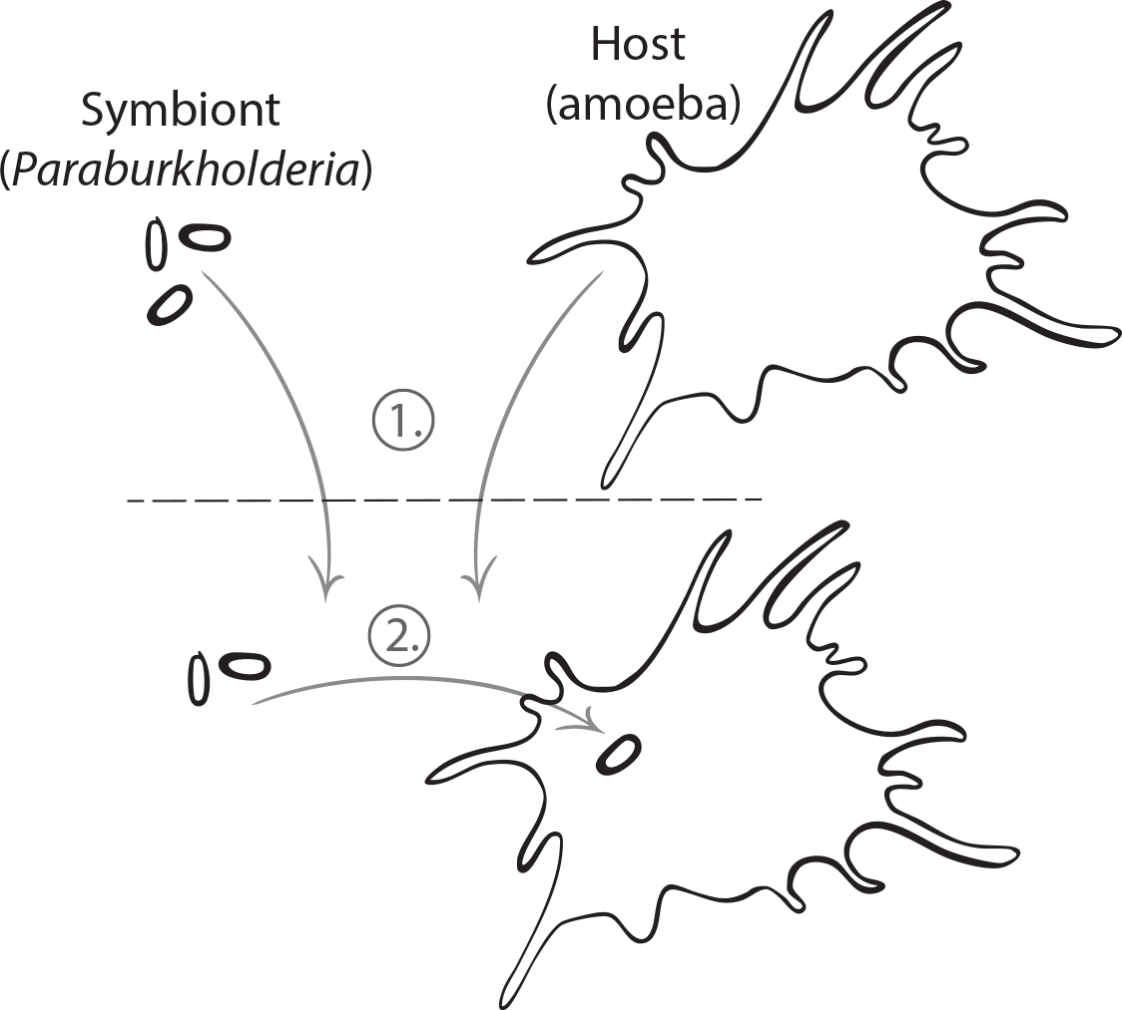
Different scales at which dispersal can be limited. Environmental acquisition of a symbiont by a host requires dispersal at two different scales (1). The symbiont and host, which in our case are *Paraburkholderia* and an amoeba, must disperse to the same local environment. Once the two organisms are in said environment (2), the symbiont must then disperse into the host intracellular environment. For our purposes, it must be ingested by the host. Dispersal can be limited at both of the scales, though their interaction is asymmetric. That is, limitation at scale 1 necessarily limits scale 2, but scale 2 can be limited even if scale 1 is not. For example, despite sufficient dispersal rates at scale 1, limitations at scale 2 may be generated by unfavorable abiotic conditions in the local environment that prevent it from achieving a sufficient density, preferential feeding on other bacteria by the host, etc. Although this figure depicts the interaction between a symbiont and a single host, dispersal at scale 2 must occur at a population level to facilitate ecologically and evolutionarily meaningful interactions over time. Therefore, dispersal at scale 2 may also be limited by stochastic loss due to ecological drift, the effects of which would be greater with lower symbiont prevalences.

Given this expanded perspective, it is important to first define the typical frameworks used to study host-symbiont dynamics in the context of general ecological theory. The concept of ecological selection is typically the most intuitive. From a symbiosis perspective, if a symbiont is either good at infecting hosts (parasitic) or provides benefits for hosts (mutualistic), ecological selection is the process by which the association rises in frequency. This can be contrasted with ecological drift, where stochasticity in reproduction and transmission results in the symbiont loss. For an extreme example, if a symbiont that would be otherwise selected for infects a single individual in a host population, the probability that it spreads to other hosts is exceedingly low. This is likely to result in the loss of the symbiont from the host population due to ecological drift (10). Therefore, a primary way to increase symbiont prevalence and combat the effects of drift is to ensure transmission fidelity (11). From the perspective of general ecological theory, transmission is analogous to dispersal. In the most general sense, dispersal refers to the density/rate at which an organism arrives in the environment of interest. For the purposes of endosymbiotic interactions, the environment of interest is the inside of a host. Therefore, symbiont transmission, whether through inheritance or environmental acquisition, fundamentally describes the same process. From this perspective, limitations on symbiont dispersal can be generated by many mechanisms. Environmentally acquired symbionts may fail to arrive in a host’s local environment at a high enough rate to mitigate probable extinction due to ecological drift (Fig. 1). Alternatively, environmentally acquired symbionts may physically disperse at a sufficient rate but are selected against for other reasons (Fig. 1). Similarly, inheritance of vertically transmitted symbionts may have a stochastic component due to intra-host dynamics. Regardless of the mechanism, the overall outcome for the host-symbiont dynamics is the same: the symbiont may not infect the host frequently enough to allow selection for the interaction to override its probable loss due to drift (10).

Despite the prevailing emphasis on selection, central theory on the evolution of endosymbiotic interactions makes implicit reference to the interplay between dispersal and selection in shaping endosymbiont distributions. The evolutionary training grounds hypothesis suggests that frequent predation by smaller and local eukaryotes (such as amoeba) selects for prokaryotes that can survive intracellular digestion, which then increases their ability to evade immune defenses and infect other hosts (12, 13). In the context of more general theory, frequent predation by local eukaryotes facilitates pre-adaptation to survive and proliferate in intra-host environments, which confers greater success when a prokaryote disperses to (infects) a new host. Under this interpretation, limitations on dispersal to host populations, rather than long-term co-evolutionary dynamics, can be a primary driver of endosymbiont distribution given significant pre-adaptation. This point has been discussed but has received relatively less attention as a framework for understanding the distribution of symbionts among hosts than long-term co-evolutionary frameworks (14).

Taken together, this framework offers several predictions regarding how symbionts are expected to be distributed across host populations, which depends on the nature and history of the interaction. First, an environmentally acquired symbiont may evolve adaptations that allow its persistence in an intra-host environment. If said symbiont encounters a host environment that is either new or too infrequent to exert a meaningful selective pressure, previously evolved adaptations may allow its persistence. If the symbiont was capable of dispersing (being transmitted) to this new host environment at a sufficiently high rate or density, it would be able to achieve a high prevalence and fitness. However, this would be limited to intra-host environments that were similar to that which it previously adapted and would decline in more dissimilar hosts. If symbiont dispersal was not limited on a broader geographic scale, this would create biogeographic patterns where symbiont diversification is unrelated to geography. However, if dispersal is limiting, one would expect patterns of symbiont diversification to be explained by their geographic distribution. In both cases, the symbiont would not be very host-specific, as it would be able to infect whatever host it encountered (at least within the limits of its pre-adaptation). These patterns are in contrast to what would be expected of a symbiont that is vertically transmitted and has a history of co-evolution and co-dispersal with a specific host. Dispersal would not be limiting in this case, and patterns of symbiont diversification would be better explained by patterns of host diversification rather than geographic distribution. Furthermore, co-evolutionary dynamics would lead to the evolution of greater host specificity, causing symbionts to show a more significant decrease in their ability to infect other hosts.

Much research on the evolution of endosymbiotic interactions tends to focus on long-term co-evolved symbionts. However, the evolutionary trajectory toward such an interaction begins with an environmentally acquired symbiont (that is typically parasitic), and descriptions of how fundamental ecological and evolutionary processes shape these earlier dynamics are limited. Here, we explore this phenomenon using the interactions between dictyostelid social amoeba hosts and their *Paraburkholderia* bacterial symbionts, which typically show parasitic tendencies (15). Because *Paraburkholderia* is environmentally acquired, this system can offer fundamental insight into the roles of pre-adaptation and dispersal limitation in shaping the distribution of more recent endosymbionts that are segregating in host populations. We first conducted a field study that showed patterns of endosymbiont diversification were highly biogeographic, suggesting an important role of dispersal limitation in shaping their distributions. We then experimentally mediated endosymbiont dispersal to host populations along a continuum of phylogenetic divergence from their focal host and found that each *Paraburkholderia* endosymbiont was able to establish and sustain a high prevalence in each host population. Underlying these interactions, we also found that the fitness consequences (for both host and endosymbiont) did not vary with phylogenetic distance from the focal host, suggesting *Paraburkholderia* endosymbionts are pre-adapted to other hosts that they do not frequently interact with.

## MATERIALS AND METHODS

### Study system

Dictyostelid social amoebae are soil-dwelling protists that prey on bacteria. When local food bacteria are depleted, thousands of individual amoebae cells aggregate to form a multicellular fruiting body that facilitates spore dispersal (16). Bacteria that have either stuck to the outside of amoeba cells or survived intracellular digestion are able to persist within these fruiting bodies, thus facilitating their co-dispersal as well (15, 17, 18). A diversity of bacterial symbionts has been found to associate with dictyostelid hosts (19). However, frequency of association, fitness consequences, intracellular dynamics, and genomic evidence of host adaptation have been predominately characterized in three *Paraburkholderia* symbionts whose native amoeba host is thought to be *Dictyostelium discoideum: Paraburkholderia agricolaris*, *Paraburkholderia bonniea*, and *Paraburkholderia hayleyella* (18, 20, 21). Therefore, here, we focused on studying the ecological processes that shape the distribution and frequency of these three symbionts in dictyostelid amoeba host communities. For experimental studies, we used *D. discoideum*, *Dictyostelium citrinum*, *Dictyostelium giganteum*, *Dictyostelium purpureum*, *Polysphondylium violaceum*, and *Cavenderia aureostipes*, because they occur sympatrically and represent a breadth of phylogenetic divergence from *D. discoideum*. For example, *D. citrinum* is a closely related sister species of the same genus, *D. giganteum* and *D. purpureum* are more distantly related members of the same genus, *P. violaceum* is of a different genus in the same family, and *C. aureostipes* is of a different order but in the same clade (22).

### Characterizing Burkholderiales symbiont prevalence and diversity in natural dictyostelid social amoeba communities

**Fig 2:**
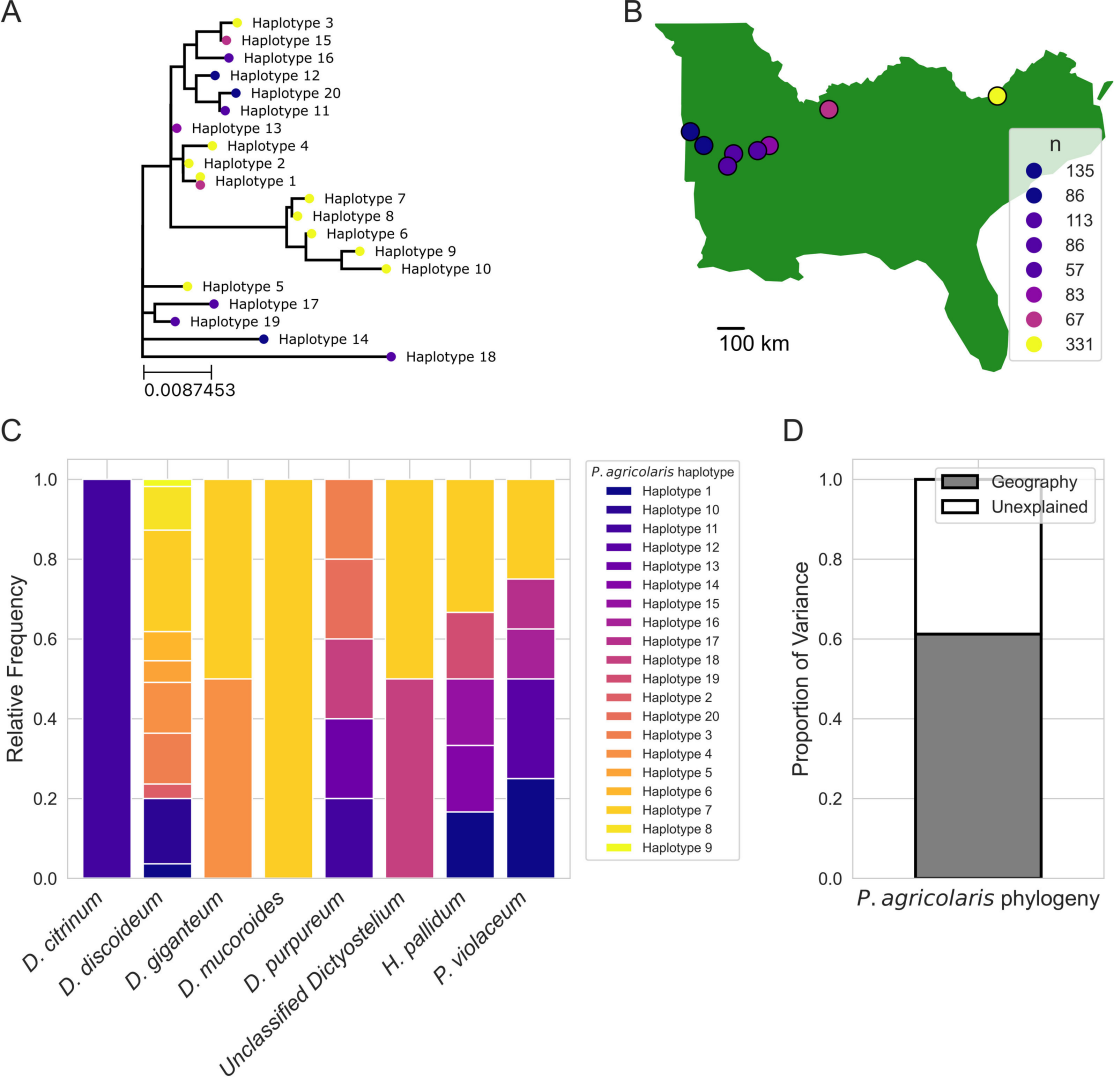
Patterns of *P. agricolaris* divergence are explained by geographic variation but not patterns of host association. (**A**) The phylogenetic tree based on LepA sequences of *P. agricolaris* haplotypes found in our surveys of natural populations. Colored circles at the tips indicate the geographic location each haplotype was found in. Note that there is a single branch with multiple (2) location points next to it, which indicates only this haplotype was found in multiple locations. (**B**) A map showing the geographic distribution of sampling locations. The legend indicates the number of amoebae isolates collected from each location. Made with Natural Earth. (**C**) Stacked bar plots showing the relative frequency of each *P. agricolaris* haplotypes in each social amoeba host. Note that the colors in this plot do not correspond to the colors in **A**, as **A** is being used to depict geographic distributions while **C** is used to depict relative frequencies in each host species. (**D**) A bar graph showing the proportion of variation in patterns of *P. agricolaris* divergence that could be explained by their geographic distribution. Patterns of host association (the frequency of *P. agricolaris* in each host populations) were included in the model but did not significantly explain any variation.

To study the distribution and frequency of *Paraburkholderia* symbionts in natural dictyostelid populations, we isolated amoeba from soil collected across the Southeastern United States (Fig. 2). We collected each soil sample from just below leaf litter on forest floors or from decaying material in rotting logs and stumps. To obtain amoeba isolates, we plated each soil sample on hay agar (Appendix section 1.1.1) no later than 96 hours after collection. After sufficient time for fruiting body formation, we identified amoeba species based on fruiting body morphology (23). Since proliferation of single amoeba cells can give rise to patches of several fruiting bodies, we collected clonal amoeba isolates from 2 to 5 sori from each patch. We suspended isolate sori in 250 µL of KK2 spore buffer (Appendix section 1.1.3) and extracted total DNA using a Chelex/proteinase K protocol described in Haselkorn et al. (18). To confirm that isolates were dictyostelid fruiting bodies and that DNA extraction was successful, we first conducted a PCR screen on all samples using dictyostelid-specific primers (D307F and D862R) that target a portion of the 18S rRNA gene (Appendix section 1.2.1) (24). We excluded samples that did not show amplification, as this indicates the DNA extraction failed, or the sampled fruiting body was not a dictyostelid. To further confirm the identity of dictyostelid isolates, we sent a subset of positive PCR products for sequencing Eurofins Genomics (Louisville, KY, USA). We then trimmed sequences using Geneious (v8) (25) and classified isolates by using the NCBI BLAST web application (https://blast.ncbi.nlm.nih.gov/Blast.cgi) to search for matches in the standard nucleotide database.

To identify the presence of Burkholderiales symbionts in each amoeba isolate (*n* = 958 across all sites considered), we first conducted a PCR screen using Burkholderiales-specific primers (Burk3F and Burk3R) that amplify a portion of the 16S rRNA gene (Appendix section 1.2.2) (26). For all positive samples, we then amplified a 780 base-pair region of the leader peptidase A (LepA) gene using LepAF and LepAR primers, which allows for greater species-level identification and haplotype (groups of genetic variants located on the same chromosome that are inherited together, equivalent to an ASV given a single gene) resolution of Burkholderiales symbionts (18). We sequenced and identified Burkholderiales symbionts as previously described for the dictyostelids. We also included the prevalence and LepA sequence data from (21) in our data set, which allowed us to capture greater geographic and phylogenetic variation in our analyses. After sequence processing, we used MEGA (v7) to align sequences (using the MUSCLE algorithm) and construct a maximum-likelihood phylogenetic tree, where our data were best described by a GTR + G + I substitution model. We used 1,000 bootstrap replicates to estimate branch support (27). The only previously described *Paraburkholderia* symbiont we found in nature was *P. agricolaris*. Therefore, we constructed a separate phylogenetic tree using only *P. agricolaris* LepA sequences, where our data were best described by a Tamura 3-parameter + G + I substitution model.

### Experimentally mediating symbiont dispersal to laboratory host populations

To experimentally facilitate the interaction between dictyostelid hosts and the focal *Paraburkholderia* symbionts, we first plated 10^5^ spores isolated from uninfected hosts with 300 µL of a mixture containing 95% *Klebsiella pneumoniae* and 5% symbiont (either *P. agricolaris*, *P. hayleyella*, or *P. bonniea*, each of which was isolated from *D. discoideum*). We used red fluorescent protein (RFP)-labeled symbiont strains because their presence in a fruiting body can be directly visualized via pink discoloration in their colonies upon culturing, and we wanted to perform subsequent microscopy to confirm intracellular infection status (described below) (15). We cultured four replica populations for each amoeba and symbiont combination. To examine whether potential symbiont infections persisted beyond initial introduction, we passaged 10 representative fruiting bodies from each host population onto a new plate containing only the host food bacteria *K. pneumoniae*. For passaging, we suspended fruiting bodies in 500 µL of KK2 spore buffer and plated 100 µL of these suspended spores with 200 µL of 1.5 OD_600_ *K. pneumoniae*. We performed two rounds of passaging (for a total of three host generations). Each generation, we measured the proportion of host fruiting bodies that contained *Paraburkholderia* (an estimate of prevalence) by culturing bacteria from 10 individual host sori, where pink colony discoloration indicated *Paraburkholderia* presence. Note that this measurement does not assess the proportion of spores infected within fruiting bodies, which is described below.

### Quantifying intracellular symbiont infection and effects on fitness

To assess fitness cost and intracellular prevalence of each symbiont-amoeba pairing, we first plated 10^5^ amoeba host spores that were isolated from uninfected amoeba fruiting bodies on SM/5 agar plates with 200 µL of *K. pneumoniae* for uninfected controls or mixed bacterial suspension consisting of 95% *K*. *pneumoniae* and 5% RFP-labeled symbiont (adjusted to 1.5 OD_600_ nm). We incubated plates at 24°C for 1 week to allow for fruiting body formation. We then collected and suspended fruiting bodies from the entire plate in KK2 spore buffer and quantified total spore productivity via hemocytometer counts. We quantified intracellular infection prevalence by sampling suspended spore samples (>1,000 spores) through a BD-C6 Flow Cytometer, gating spores, and quantifying percent of RFP-positive spores via PE-A intensity histograms, where uninfected samples were used to establish fluorescent and non-fluorescent spore boundaries. To visualize infections via confocal microscopy, we suspended spores from developed fruiting bodies in KK2 buffer supplemented with 1% calcofluor, placed them onto glass-bottom culture dishes, and overlaid 2% agarose. We used an Olympus Fluoview FV1000 confocal microscope equipped with a Plan Apo Oil 60×/1.4 NA objective to acquire images, where we used the DAPI channel to visualize calcofluor-stained structures (pseudo-colored gray) and the Cy3 channel to visualize RFP (pseudo-colored red). We acquired Z-stacks at 0.5 µm intervals at a resolution of 1,024 × 1,024 pixels and used Fiji (NIH; imagej.net/Fiji) to process images, including generation of single slices and z-projections (15, 18).

### Statistics

To quantify the importance of host association frequency and geography in explaining patterns of *P. agricolaris* diversification, we performed a distance-based redundancy analysis using the *capscale* function from the *vegan* R library (28). First, we used the *cophenetic.phylo* function from the *ape* R library to transform the LepA *P. agricolaris* phylogeny into a pairwise phylogenetic distance matrix (29) and the *distm* function from the *geosphere* R library to calculate the geographic (Haversine) distance matrix between sample location (30). We then calculated the proportion of variance explained by geographic dissimilarity and host association frequency using the *varpart* function from the *vegan* R library (28). In addition to a distance-based redundancy analysis, we also performed a Mantel test (with 999 permutations) to quantify the correlation between phylogenetic distance and geographic distance between *P. agricolaris* haplotypes (28).

To investigate the relationship between phylogenetic distance from *D. discoideum* and fitness effects associated with symbiont infection (as estimated from variation in spore production), we first used the previously described dictyostelid 18S sequences to create a phylogenetic tree of the social amoebae species used in our study, where our data were best described by a GTR + G + I substitution model. We then quantified the cophenetic distance (which we refer to as phylogenetic distance for simplicity) from each species to *D. discoideum* using the *cophenetic.phylo* function from the *ape* R library (29). To model the relationship between phylogenetic distance from *D. discoideum* and symbiont intracellular prevalence, we fit a generalized linear model using the *glm* R function with a quasibinomial distribution to account for observed overdispersion (31). To model the relationship between phylogenetic distance from *D. discoideum* and amoeba fitness effects, we fit a linear model using the *lm* R function (31).

## RESULTS

### Biogeographic patterns of *Paraburkholderia* symbionts are strong in dictyostelid social amoebae populations

Our natural survey revealed 20 *P*. *agricolaris* haplotypes across all sampled locations (Fig. 2A). To begin understanding the relative importance of dispersal limitation and specific associations in shaping the distribution of *Paraburkholderia* symbionts among social amoeba hosts, we modeled patterns of *Paraburkholderia* phylogenetic divergence (Fig. 2A) as a function of their geographic distribution (Fig. 2B) and frequencies in different host populations (Fig. 2C). We found that the geographic distribution of *P. agricolaris* (the only previously described symbiont found) explained 61.216% of their patterns of phylogenetic divergence (*F* = 3.1589, *P* = 0.038) (Fig. 2D). However, specific patterns of host association did not significantly explain any of the patterns in *P. agricolaris* phylogenetic divergence (*F* = 0.9910, *P* = 0.507), and we did not find any covariance between geographic distributions and specific patterns of host association (Fig. 2D). Furthermore, we found a weak but significantly positive correlation between geographic distance and phylogenetic distance between *P. agricolaris* haplotypes (r_M_ = 0.1826, *P* = 0.012), suggesting a combination of isolation by distance as well as more localized isolation.

### *Paraburkholderia* symbionts maintain stable associations with a diversity of social amoebae hosts when dispersal is mediated

**Fig 3:**
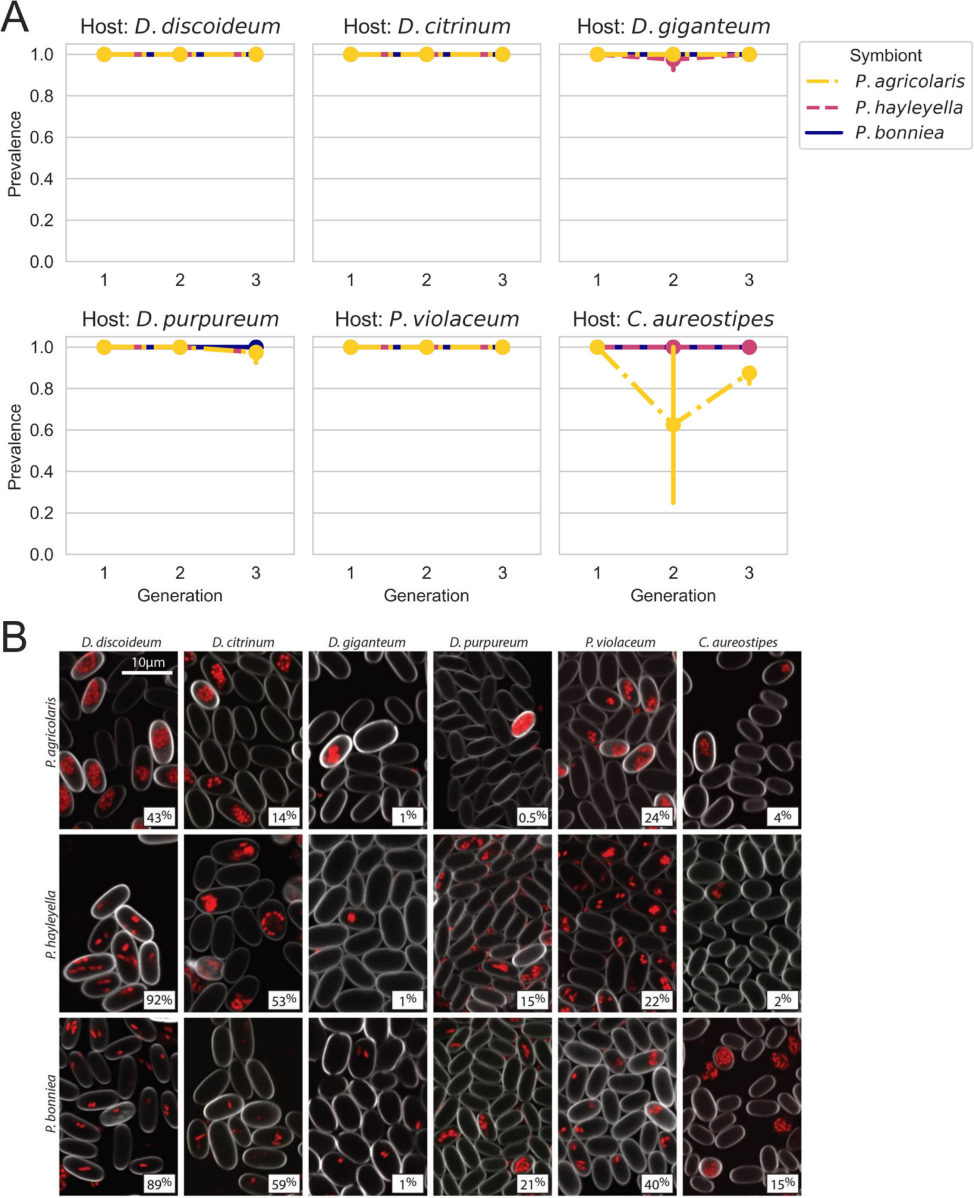
*Paraburkholderia* symbionts form stable and intracellular interactions with a variety of social amoeba hosts upon experimentally mediated dispersal to their local environment. (**A**) The prevalence of *Paraburkholderia* symbionts in host fruiting bodies for each amoeba host species. Note that this prevalence represents the proportion of fruiting bodies infected, not the proportion of spores within fruiting bodies infected. (**B**) Confocal microscopy images of developed sori contents from indicated amoeba species following exposure to RFP-labeled *Paraburkholderia* symbionts. Images show that each *Paraburkholderia* can intracellularly infect (to variable degrees) each host species. Quantitative inferences regarding intracellular infection prevalence were done using the more high-throughput flow cytometry and are later described.

Our survey of natural social amoebae populations suggests an important role of dispersal limitation in shaping the distribution of *Paraburkholderia* endosymbionts among hosts. Therefore, the following prediction would be that when dispersal is experimentally mediated, *Paraburkholderia* symbionts should be able to establish and persist in various social amoebae populations. To test this prediction, we experimentally introduced the three previously described *Paraburkholderia* symbionts into six different social amoebae populations and measured their prevalence over three generations. Symbiont prevalence remained at or near 100% across generations for most host-symbiont pairings (Fig. 3A). The only exception was *P. agricolaris* in *C. aureostipes* hosts, which still maintained 60%–80% prevalence (Fig. 3A). This showed that each symbiont was able to stably infect each host population at a high prevalence. Because *Paraburkholderia* symbionts can intracellularly and extracellularly infect amoeba hosts, we used RFP-labeled symbionts and confocal microscopy to confirm that symbionts were intracellularly infecting hosts. This showed that each symbiont was able to infect the spores of each host (Fig. 3B).

### Intracellular infection frequency and fitness consequences of *Paraburkholderia* symbionts do not vary with their host’s phylogeny

**Fig 4:**
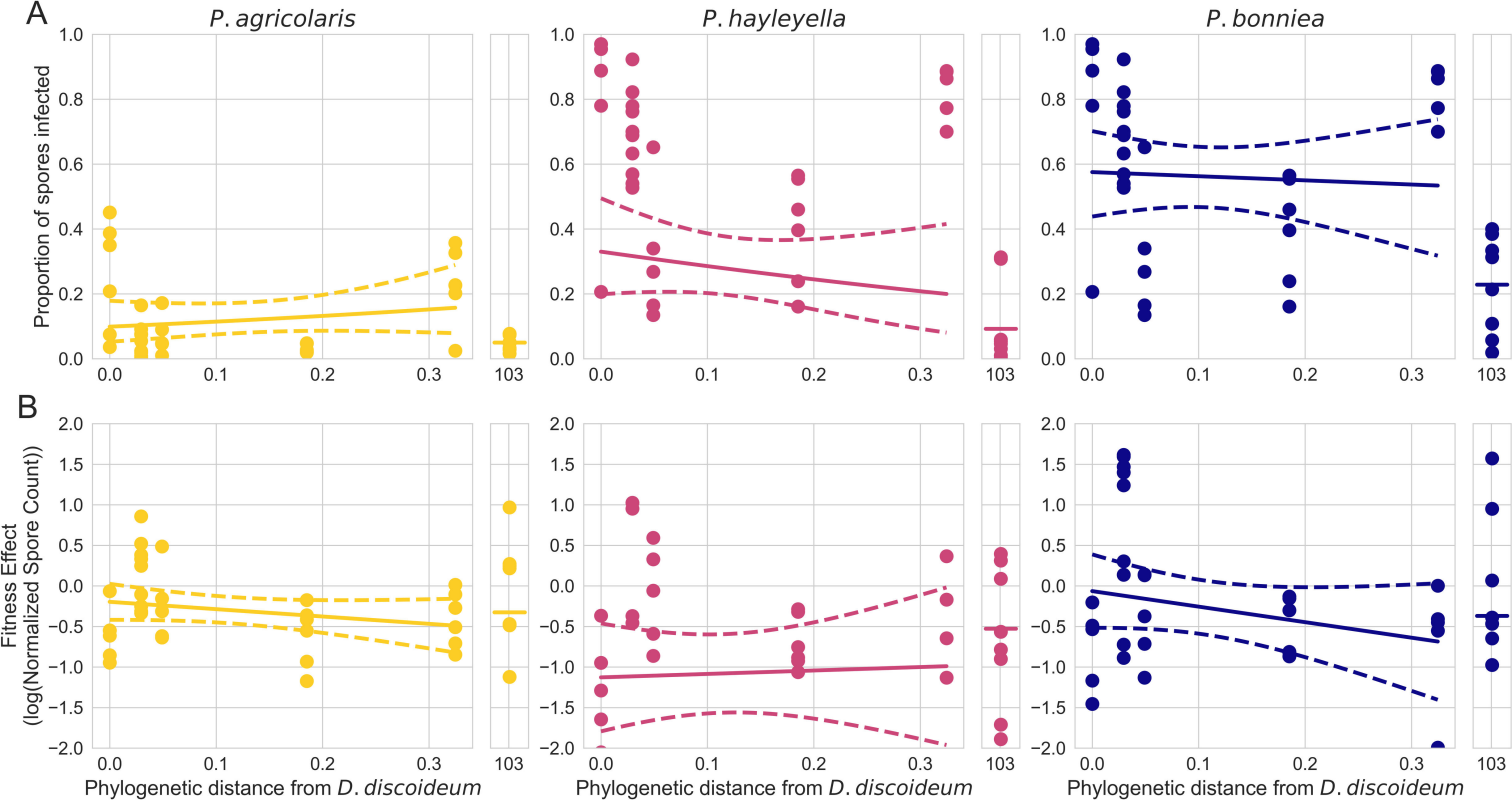
The fitness effects and intracellular infection prevalence of host-symbiont interactions do not vary with increasing phylogenetic distance from their focal host *D. discoideum*. (**A**) The proportion of host spores symbionts was able to infect as a function of phylogenetic distance from *D. discoideum*. (**B**) Log-transformed normalized spore counts as a function of phylogenetic distance from *D. discoideum*. Note that values less than 0 indicate that infected hosts produced fewer spores than an uninfected control. For each panel, solid lines represent the fitted model predictions, dashed lines represent the 95% confidence interval, and points represent the observed data.

Our previous experiment suggests that given sufficient dispersal, each *Paraburkholderia* symbiont could stably infect each amoeba species, so we were then interested in the potential role of pre-adaptation in facilitating this dynamic. If the interactions between a symbiont and a host facilitated significant pre-adaptation, one would predict that the fitness consequences of the interaction (for both host and symbiont) in other hosts should be comparable to that of the focal host. Consistent with this prediction, we found that the intra-host fitness of each *Paraburkholderia* symbiont (measured by proportion of host spores infected) did not vary with the phylogenetic distance from their presumed focal host *D. discoideum* (*P* > 0.2013; see Appendix section 2.1 for full model summaries) (Fig. 4A). However, we also found that each symbiont exhibited a reduced ability to infect host spores in a highly diverged host species (*C. aureostipes*), suggesting limitations on pre-adaptation (Fig. 4A). Likewise, we found that the host fitness consequences (measured by host spore production) of association with each symbiont did not vary with phylogenetic distance from the presumed focal host *D. discoideum* (*P* > 0.206; see Appendix section 2.2 for full model summaries) (Fig. 4B).

### Social amoebae interact with a diverse range of Burkholderiales symbionts in natural populations

**Fig 5:**
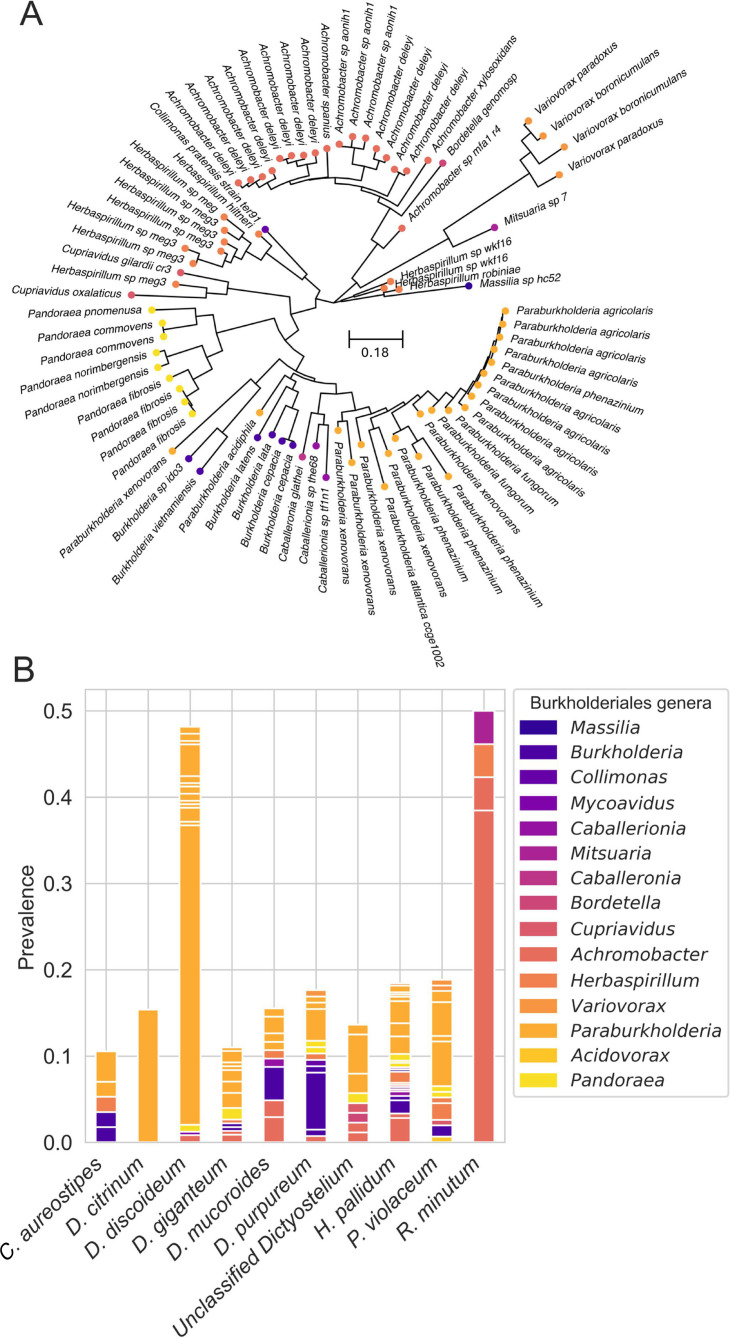
Dictyostelid social amoeba associate with diverse species of Burkholderiales in nature. (**A**) A phylogeny based on the LepA gene of Burkholderiales symbionts found associated with each sampled social amoeba host. Across all sampled amoeba hosts, we identified 79 unique LepA haplotypes from 15 Burkholderiales genera. (**B**) The prevalence of each Burkholderiales haplotype within their respective amoeba host species. Each bar corresponds with a host species, and the *y*-axis shows the prevalence of symbionts within said host species. Note that some bars contain subdivisions, which indicate that multiple LepA haplotypes were found within the corresponding genus.

During our screenings of natural populations, we found that dictyostelid social amoebae harbor a diverse range of symbionts within the Burkholderiales order. Specifically, we found 79 unique LepA haplotypes spanning 15 Burkholderiales genera across all host species (Fig. 5A). Host species varied in the overall frequency at which they harbored Burkholderiales symbionts. The total prevalence in most host species is between 10% and 20% (Fig. 5B). However, Burkholderiales prevalence was over twice as high in *D. discoideum* and *Dictyostelium minutum* (Fig. 4B).

## DISCUSSION

Understanding how ecological processes shape the frequency and distribution of endosymbionts that are segregating in host populations is fundamental for understanding the evolution of endosymbiotic interactions. Here, we found evidence that dispersal limitation plays an important role in shaping the distribution of endosymbioses between social amoeba and their *Paraburkholderia* symbionts. Our survey of natural social amoeba communities showed that patterns of *P. agricolaris* (the only previously described endosymbiont found in our natural survey) diversification were predominately explained by geographic dissimilarity, suggesting the interaction experiences a significant degree of dispersal limitation (Fig. 2). We then experimentally mediated symbiont dispersal to host environments to test the importance of dispersal limitation in determining the distribution of these interactions. We found that each previously described *Paraburkholderia* symbiont was able to stably and intracellularly infect each host species at a high prevalence (Fig. 2). Furthermore, host-selective dynamics (fitness consequences of the interaction) did not vary with increasing phylogenetic distance from the focal host *D. discoideum* (Fig. 3). Finally, we also found that dictyostelid social amoebae tend to associate with a wide diversity of Burkholderiales bacteria (Fig. 4).

Overall, our findings suggest that the endosymbiotic interactions between dictyostelid amoeba and *Paraburkholderia* symbionts are shaped by a high degree of dispersal limitation, where pre-adaptation facilitates their interactions upon occupation of a shared environment. Therefore, it is possible that limitations on symbiont or host dispersal, rather than specific symbiont functions, explain the variable frequency and often absence of these interactions in natural populations (18, 21). Laboratory studies have described several possible functions of *Paraburkholderia* symbionts for their amoeba hosts, which include toxin resistance (32) and facilitate bacterial food carriage (15). However, these symbionts generally have negative fitness consequences (which our findings recapitulated), and field studies have not found evidence that these laboratory-described functions are relevant in natural contexts (15, 18, 21). Therefore, it seems unlikely that selective benefits conferred by previously suggested symbiont functions have played an important role in the ecological or evolutionary dynamics of these interactions. Rather, our findings suggest a simpler model where dictyostelid amoebae happen to ingest *Paraburkholderia* symbionts that are in their local environment, and said symbionts are generally pre-adapted to survive ingestion by amoeba and confer some fitness cost. Our finding that a wide diversity of Burkholderiales symbionts seems to associate with dictyostelid hosts suggests this model may apply generally to other symbionts besides those that have been previously described. However, we suggest this with the caveat that we did not discern whether these other symbionts could actually persist within the host.

Considering dispersal limitation has the potential to significantly expand our understanding of the evolutionary dynamics of these amoebae-bacteria endosymbioses. For example, even if previously described symbiont functions do occur in nature, their relevance may only be sparsely realized by a small portion of the host population. In other words, dispersal limitation could limit the selective spread of potential beneficial associations, which would also be consistent with the highly variable prevalence seen in nature (18, 21). It is easy to consider a scenario where the frequency at which a symbiont appears in association with a host is significantly influenced by the frequency at which it disperses to said host’s environment (or vice versa). Theory suggests that at low frequencies, the symbiont would be more subject to ecological drift and would have a greater likelihood of being stochastically lost (7, 10). Therefore, deterministic outcomes from local factors associated with dispersal (e.g., such as alleviated competition, which some evidence suggests may be at play in this system) (33) may be hindered due to limitations on dispersal. Our study is limited in that we manipulated symbiont dispersal at a fixed density. However, future studies that examine symbiont colonization/loss in amoeba populations as a function of their immigration density could directly test this hypothesis, which would give useful insight into the incredibly variable symbiont prevalence seen in nature.

Our study is not able to conclude whether limitations on host dispersal, symbiont dispersal, or both are responsible for the observed patterns of dispersal limitation of the interaction. There is evidence that natural dictyostelid populations are dispersal-limited, as genetic differentiation between *D. discoideum* populations has been observed at narrow and broad geographic scales (34, 35). However, less is known of population structure in other dictyostelid species. Symbionts could also experience limitations on dispersing to host environments. For example, abiotic conditions impact the growth and distribution of Burkholderiales bacteria, including the symbionts studied here (20, 36). Therefore, abiotic conditions could selectively limit the distribution of symbionts, which could in turn limit their dispersal to host environments. It is likely that both factors are at play, but further work would be needed to establish their relative contributions.

Consistent with previous studies, we also found that *Paraburkholderia* symbionts appear pre-adapted to survive and proliferate in a variety of dictyostelid hosts (37). Furthermore, we found that the fitness effects of these interactions did not decline with increasing phylogenetic distance from the focal host *D. discoideum*. This is generally consistent with a previous study that found a variety of *P. bonniea* symbiont strains did not have different consequences on fitness when paired with native and novel host genotypes (38). Given the growing body of evidence suggesting a lack of specificity in these interactions, it is possible that *D. discoideum* is not necessarily the focal host as previously suggested. The genomes of *P. bonniea* and *P. hayleyella* symbionts show significant signatures of erosion due to long-term occupation of intracellular host environments (20). However, natural surveys have consistently found that they do not frequently associate with *D. discoideum* (18, 21), and our findings now suggest that they do not frequently associate with other common dictyostelid species either. Therefore, it is possible that their primary host is not *D. discoideum* or even another closely related species. Rather, it is possible that previous recordings of these associations in nature are simply the product of infrequent dispersal from an alternative focal host’s population into *D. discoideum* populations. Conversely, the *P. agricolaris* genome and physiology are more similar to other free-living *Paraburkholderia* species and also form the most frequent associations with dictyostelid hosts. Therefore, it is possible that its general patterns of association with dictyostelid hosts are the product of slightly alleviated dispersal limitation relative to the other two symbionts, which could be mediated by its greater competitiveness when free-living (20). Alternatively, it is possible that *P. agricolaris* is more competitive in the intra-host environment than either of its counterparts. Therefore, during co-infection, *P. agricolaris* may be able to outcompete *P. bonniea* or *P. hayleyella*, as seen in some of the various Burkholderiales symbionts of bean bugs (39). This mechanism warrants testing in the future but seems unlikely because *P. agricolaris* is predicted to be both more competitive in the soil environment and less able to proliferate within hosts (Fig. 4A).

More generally, the role that dispersal plays in shaping the frequency of host-symbiont interactions is fundamental for understanding their evolutionary dynamics. However, it remains unclear if dispersal limitation is common among host-microbe interactions. For example, biogeographic patterns are common in many systems, ranging from sea sponges to legumes, but further experimentation is needed to establish whether such patterns are driven by dispersal limitation or local selective pressures (40–42). Likewise, there can also be significant variation in patterns of dispersal limitation within a system, as seen in several bacterial symbionts of marine bivalves (43). Nonetheless, in many different systems, there are often discrepancies between symbiont functions described in laboratory studies and the apparent relevance of said functions in nature (21, 44). There is a growing appreciation for the role of selection in explaining these complexities, where the net benefit or detriment of a symbiotic interaction changes depending on what stressors are at play in the environment at a given time (45). Our findings suggest it is also important to understand the role of dispersal in limiting or facilitating the spread of potential functions. For example, if a symbiotic interaction becomes beneficial in only a portion of a population that is dispersal limited, it would be more difficult for this benefit to be generalized to the whole population. This issue compounds when considering a given interaction may only be beneficial between hosts and symbionts of particular genetic backgrounds or in particular environments. Therefore, future studies on the interplay between dispersal and other ecological processes have the potential to significantly improve our understanding of the evolution of endosymbioses.

## Data Availability

All sequences generated for this paper have been deposited in GenBank and are available under the accession numbers PX051017 to PX051095. All code and data associated with this manuscript can be found at https://github.com/gabe-dubose/paraburk_dispersal.

## SUPPLEMENTAL MATERIAL

Data Set S1 aem.01615-25-s0001.csv All collection information.

Supplemental material aem.01615-25-s0002.pdf Supplemental methods and supporting results.

ASM does not own the copyrights to Supplemental Material that may be linked to, or accessed through, an article. The authors have granted ASM a non-exclusive, world-wide license to publish the Supplemental Material files. Please contact the corresponding author directly for reuse.
