# Supplementary material for: The roles of dispersal limitation and pre-adaptation in shaping Paraburkholderia endosymbiont frequencies in social amoeba communities
Source: Appl Environ Microbiol. 2025 Nov 7;91(12):e01615-25. doi: 10.1128/aem.01615-25 (PMC12724192; doi:10.1128/aem.01615-25)
Supplement: Supplemental material — Supplemental methods and supporting results. [file aem.01615-25-s0002.pdf]

Appendix for:
The roles of dispersal limitation and pre-adaptation in shaping Paraburkholderia endosymbiont frequencies in social amoeba communities

Contents

1 Extended Methods **2**
1.1 Culture media and buffers 2
1.1.1 Hay agar 2
1.1.2 SM/5 agar 2
1.1.3 KK2 spore buffer 2
1.2 PCR primers and protocol 2
1.2.1 Dictyostelid 2
1.2.2 Burkholderiales 3
1.2.3 LepA 3
1.2.4 Protocol 3
1.3 Amoeba and symbiont strain information 3

2 Supporting results **4**
2.1 Host relatedness 4
2.2 Model summaries for spore infection prevalence as a function of phylogenetic distance from *D. discoideum* 4
2.2.1 *P. agricolaris* 4
2.2.2 *P. hayleyella* 4
2.2.3 *P. bonniea* 4
2.3 Model summaries for host fitness response to infection as function of phylogenetic distance from *D. discoideum* 5
2.3.1 *P. agricolaris* 5
2.3.2 *P. hayleyella* 5
2.3.3 *P. bonniea* 5
2.4 Distribution of host taxa across sampling locations 6
2.5 Correlation between *P. agricolaris* phylogenetic distance and geographic distance 7

1 Extended Methods

1.1 Culture media and buffers

1.1.1 Hay agar

Ingredients (per liter):

- 15g hay
- 1.5L deionized H_2O
- 1.5g KH_2PO_4
- 0.62 g Na_2HPO_4
- 15g agar

Preparation:
Boil water in solution until infused. Filter hay out of solution and add each ingredient. Autoclave and pour before solution solidifies.

1.1.2 SM/5 agar

Ingredients (per liter):

- 1L deionized H_2O
- 2g glucose
- 2g BactoPeptone
- 2g yeast extract
- 0.2g $MgCl_2$
- 1g K_2HPO_4
- 1.9g KH_2PO_4
- 15g agar

Preparation:
Mix each ingredient, autoclave, and pour before solution solidifies.

1.1.3 KK2 spore buffer

Ingredients (per liter):

- 1L deionized H_2O
- 0.67g K_2HPO_4
- 2.25g KH_2PO_4

Preparation:
Mix ingredients, autoclave.

1.2 PCR primers and protocol

1.2.1 Dictyostelid

D307F: 5'-GTTTGGCCTACCATGGTTGTAA-3'
D862R: 5'-GGGSGTTCATATTGGGGCG-3'

1.2.2 Burkholderiales

Burk3F: 5'-CTGCGAAAGCCGGAT-3'
Burk3R: 5'-TGCCATACTCTAGCYYGC-3'

1.2.3 LepA

LepAF: 5'-CTSATCATCGAYTCSTGGTTCG-3'
LepAR: 5'-CGRTATTCCTTGAACTCGTARTCC-3'

1.2.4 Protocol

95 C (30sec), 55 C (30s), 72 C (45s) x 35 cycles

1.3 Amoeba and symbiont strain information

Host strain summaries

Table S1: Host strains and assay summaries. 'x's denote that the strain was used in the corresponding assay.

*G*enus	Species	Strain	Fitness and infection prevalence	Confocal Microscopy
Cavenderia	aureostipes	Cav1	x	
Cavenderia	aureostipes	Cav3	x	x
Dictyostelium	citrinum	Cit1	x	x
Dictyostelium	citrinum	Cit3	x	
Dictyostelium	discoideum	QS18	x	
Dictyostelium	discoideum	QS864	x	x
Dictyostelium	giganteum	tab10B1	x	
Dictyostelium	giganteum	tab113c	x	x
Dictyostelium	purpureum	33B	x	
Dictyostelium	purpureum	H10	x	x
Polysphondylium	violaceum	H20	x	x
Polysphondylium	violaceum	pvsd1	x	
Polysphondylium	violaceum	TV15	x	
Polysphondylium	violaceum	TV15	x	

Symbiont strain summaries

Table S2: Symbiont strains and assay summaries. 'x's denote that the strain was used in the corresponding assay.

*G*enus	Species	Strain	Fitness and infection prevalence	Confocal Microscopy
Paraburkholderia	agricolaris	Pa70-rfp	x	x
Paraburkholderia	bonniea	Pb859-rfp	x	x
Paraburkholderia	hayleyella	Ph11-rfp	x	x

Table S3: Host isolation locations

*H*ost	Isolation Location
D. discoideum	Mountain Lake, VA
D. giganteum	Mountain Lake, VA
D. citrinum	Woolly Hollow State Park, AR
D. purpureum	Mountain Lake, VA
P. violaceum	Mountain Lake, VA
C. aerostipes	Wooly Hollow State Park, AR

Table S4: Symbiont isolation hosts

*S*ymbiont	Host
P. agricolaris	D. discoideum
P. hayleyella	D. discoideum
P. bonniea	D. discoideum

2 Supporting results

2.1 Host relatedness

2.2 Model summaries for spore infection prevalence as a function of phylogenetic distance from *D. discoideum*

2.2.1 *P. agricolaris*

Table S5: Quasi-binomial model summary

*C*oefficients	Estimate	Std. Error	t	$Pr(>\|t\|)$	95% CI
Intercept	0.099	0.3497	-6.320	5.72e-07	0.05 - 0.17
Distance from *D. discoideum*	0.83671519	1.8480	0.884	0.384	0.11 - 0.99

2.2.2 *P. hayleyella*

Table S6: Quasi-binomial model summary

*C*oefficients	Estimate	Std. Error	t	$Pr(>\|t\|)$	95% CI
Intercept	0.33	0.3501	-2.021	0.0526	0.20 - 0.49
Distance from *D. discoideum*	0.11	2.2986	-0.912	0.3695	0.001 - 0.91

2.2.3 *P. bonniea*

Table S7: Quasi-binomial model summary

*C*oefficients	Estimate	Std. Error	t	$Pr(>\|t\|)$	95% CI
Intercept	0.56	0.2815	1.078	0.290	0.44 - 0.70
Distance from *D. discoideum*	0.37	1.9088	-0.271	0.788	0.014 - 0.96

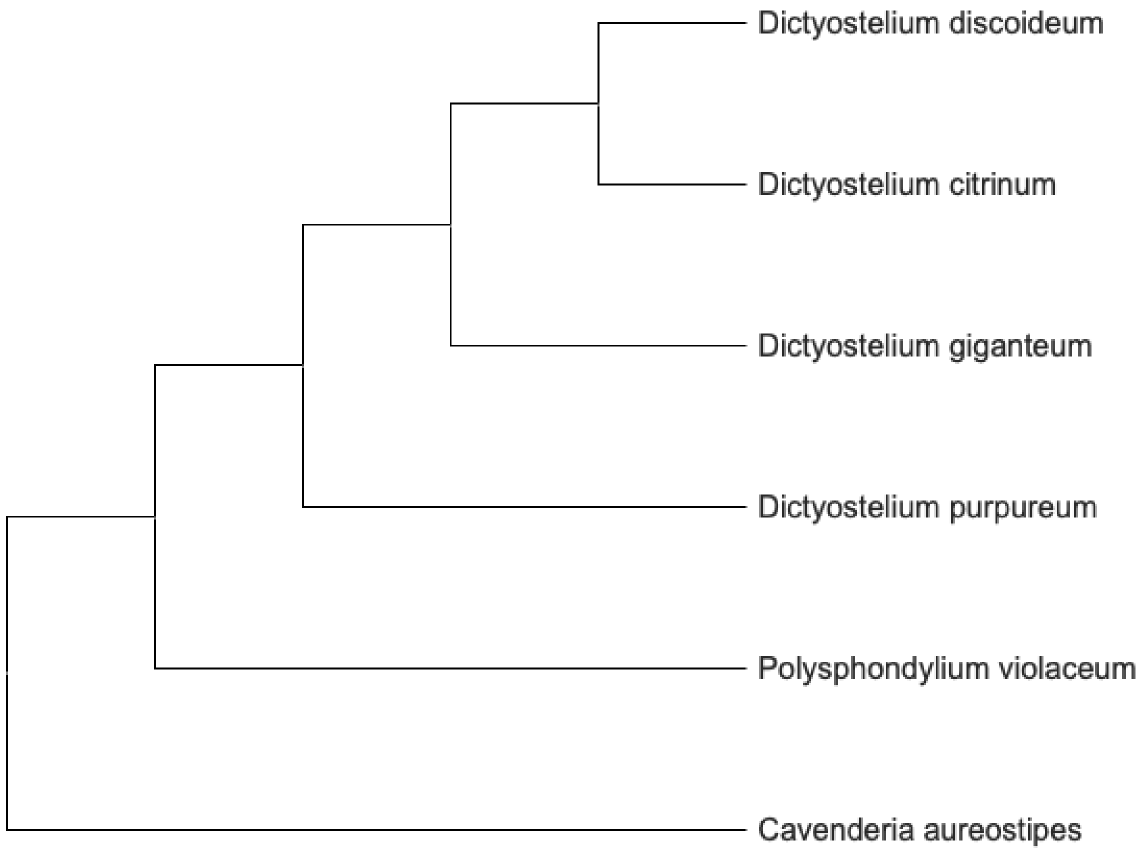

Figure S1: Cladogram showing host relatedness. Note that a we chose to display a cladogram (arbitrary branch lengts) because the divergence of *C. aureostipes* is orders of magnitude greater than divergence between other clades. Therefore, in a phylogeny with meaningful branch lengths, this scaling makes it difficult to discern relatedness between other hosts.

2.3 Model summaries for host fitness response to infection as function of phylogenetic distance from *D. discoideum*

2.3.1 *P. agricolaris*

Table S8: Quasi-binomial model summary

Coefficients	Estimate	Std. Error	t	$Pr(>\|t\|)$	95% CI
Intercept	-0.1968	0.1133	-1.737	0.0926	-0.43 - 0.035
Distance from *D. discoideum*	-0.9041	0.6920	-1.306	0.2013	-2.32 - 0.51

2.3.2 *P. hayleyella*

2.3.3 *P. bonniea*

Table S9: Quasi-binomial model summary

$Coefficients$	Estimate	Std. Error	t	$Pr(>\|t\|)$	95% CI
Intercept	-1.1282	0.3396	-3.322	0.00243	-1.82 - -0.43
Distance from *D. discoideum*	0.4243	2.0425	0.208	0.83690	-3.753070 - 4.6015832

Table S10: Quasi-binomial model summary

$Coefficients$	Estimate	Std. Error	t	$Pr(>\|t\|)$	95% CI
Intercept	-0.06471	0.23094	-0.280	0.781	-0.54 - 0.41
Distance from *D. discoideum*	-1.91432	1.48	-1.3	0.206	-4.94 - 1.12

2.4 Distribution of host taxa across sampling locations

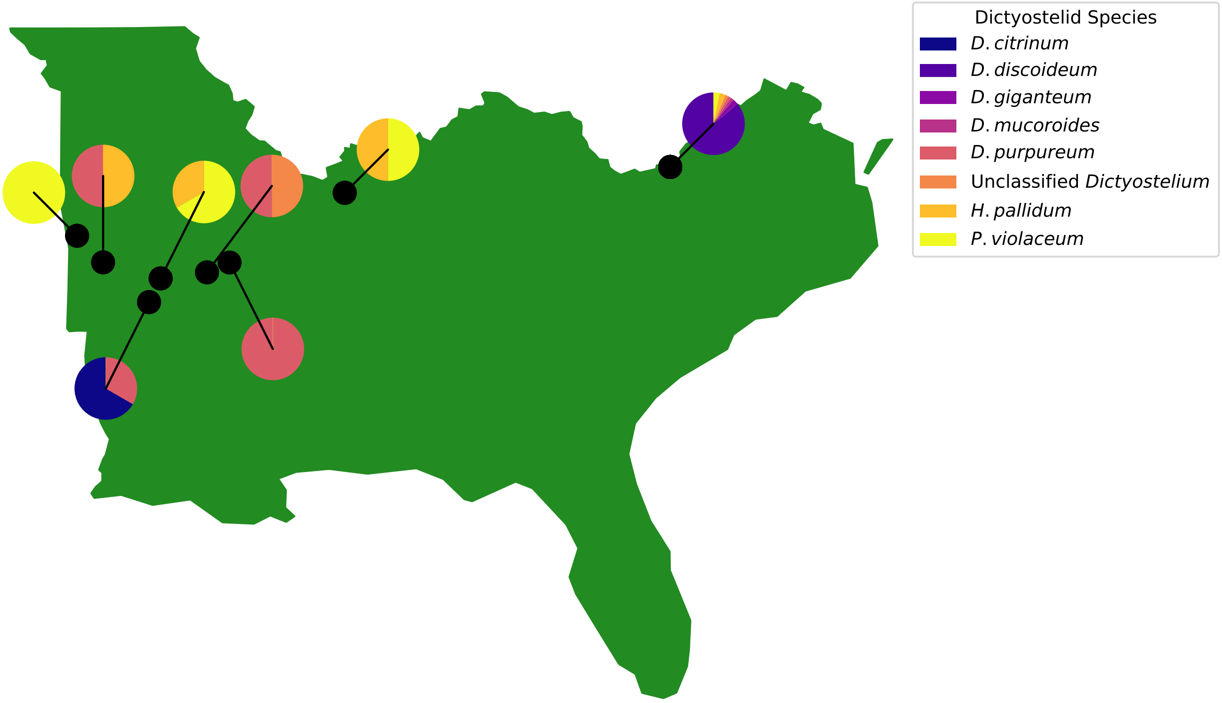

Figure S2: Distribution of host taxa across sampling locations.

2.5 Correlation between *P. agricolaris* phylogenetic distance and geographic distance

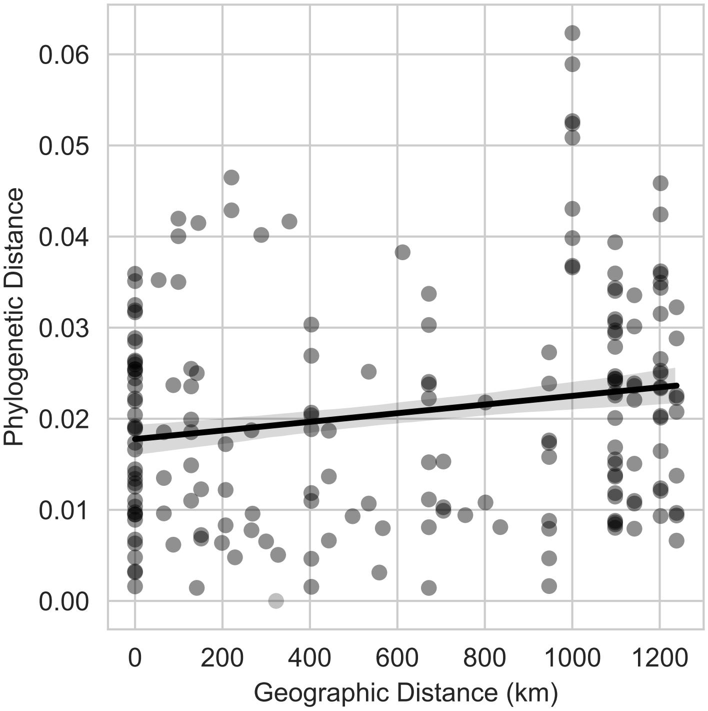

Figure S3: Distribution of host taxa across sampling locations.
